# Complement mediated thrombotic microangiopathy after liver transplantation in combination with a novel C6 variant of uncertain significance

**DOI:** 10.3389/fimmu.2026.1774160

**Published:** 2026-06-18

**Authors:** Nicola Sariye Pollmann, Lisa Hauptmann, Martin Busch, Lukas Pollmann, Andrea Tannapfel, Oliver Rohland, Aladdin Ali Deeb, Falk Rauchfuß, Utz Settmacher, Felix Dondorf

**Affiliations:** 1 Department of General, Visceral, and Vascular Surgery, Jena University Hospital, Jena, Germany; 2 Department of Internal Medicine III, Jena University Hospital, Jena, Germany; 3 Institute of Pathology, Ruhr-University Bochum, Bochum, Germany

**Keywords:** C5 inhibitor, complement system, eculizumab, liver transplantation, ravulizumab, thrombotic microangiopathy

## Abstract

Thrombotic microangiopathies (TMAs) encompass a spectrum of severe pathological conditions mostly characterized by hemolytic anemia, microvascular thrombosis with organ failure, and thrombocytopenia. The etiological spectrum of TMA is diverse, with a notable association in transplant recipients, particularly linked to the administration of calcineurin inhibitors (CNI) and due to perioperative stress factors. The clinical case presented concerns the occurrence of complement-mediated TMA (cTMA) in a recipient following liver transplantation (LT) who had previously been diagnosed with autoimmune hepatitis (AIH). The patient received total plasma exchange, followed by treatment with the anti-complement factor C5 antibody ravulizumab shortly after diagnosis. Significant improvement in the patient’s clinical condition and a decline in renal impairment was observed. Subsequently, dialysis therapy could be discontinued. In addition, an enhancement in liver functionality was detected. The diagnosis of cTMA was undoubtedly attributable to a multifactorial cause. Genetic testing identified variants involving complement factor H-related protein 5 (CFHR5) and complement component 6 (C6). The identified C6 variant was classified as a variant of uncertain significance (VUS), and its pathogenic relevance in complement-mediated TMA remains unclear. Notably, a sustained clinical response was documented six months after starting ravulizumab treatment, highlighting the complexities of managing complement-mediated disorders and the potential for durable responses using the therapy at the earliest possible time.

## Introduction

Complement-mediated TMA (cTMA) is a form of TMA that poses a critical challenge in the context of major surgical procedures, such as liver transplantation (LT), primarily due to its association with severe renal complications. Moreover, cTMA poses a substantial threat to transplanted organs and may lead to graft failure, thus significantly impacting patient outcomes.

TMA is characterized by endothelial dysfunction and microvascular thrombosis leading to hemolysis, thrombocytopenia and acute kidney failure. There are primary forms of TMA such as hemolytic uremic syndrome (HUS) and complement-mediated HUS (formerly atypical HUS/aHUS) which corresponds to cTMA. Multiple potential etiologies of TMA after LT (TA-TMA) were identified, with calcineurin inhibitor (CNI) toxicity as the leading cause, followed by perioperative endothelial injury, or infectious complications (1, 2).

Differentiating between these entities is clinically critical, as it directly impacts therapeutic decision-making, particularly with regard to modification of immunosuppressive therapy versus initiation of targeted complement inhibition (1).

Complement dysregulation in cTMA is frequently associated with genetic alterations affecting key regulatory proteins of the alternative pathway, including complement factor H (CFH) and CFH-related proteins (2). However, the interpretation of genetic findings remains challenging, as many detected variants may represent susceptibility factors or VUS rather than definitively pathogenic alterations.

Beyond its established role in host defense, the complement system has increasingly been recognized as a central mediator of ischemia-reperfusion injury and endothelial activation following solid organ transplantation (3, 4). In the setting of LT, perioperative endothelial injury and glycocalyx disruption may promote activation and amplification of the alternative complement pathway on microvascular surfaces, thereby contributing to endothelial dysfunction and microvascular injury (5, 6).These processes are increasingly recognized as important contributors to transplant-associated thrombotic microangiopathy and post-transplant organ dysfunction (7).

Recent studies have identified mutations in complement regulatory proteins, such as complement factor H, as significant contributors to the pathogenesis of cTMA, impacting treatment responses and long-term outcomes (8–10). In this context, genetic predispositions are frequently subclinical yet have the potential to manifest in clinical contexts due to acute stressors including pregnancy, or major surgery. However, the interpretation of genetic variants remains challenging, as many identified variants may represent susceptibility factors rather than definitively pathogenic mutations, and clinical manifestation often requires additional triggering factors.

In addition to those genetic predispositions, the pathogenesis of cTMA following LT is not yet fully understood. In the domain of LT, there have been a number of cases where patients with predominant TMA underwent a simultaneous liver and kidney transplant (10, 11). One case reported the occurrence of TMA five weeks following LT and subsequent treatment with eculizumab, based on management in kidney transplantation (12).

**Figure 1:**
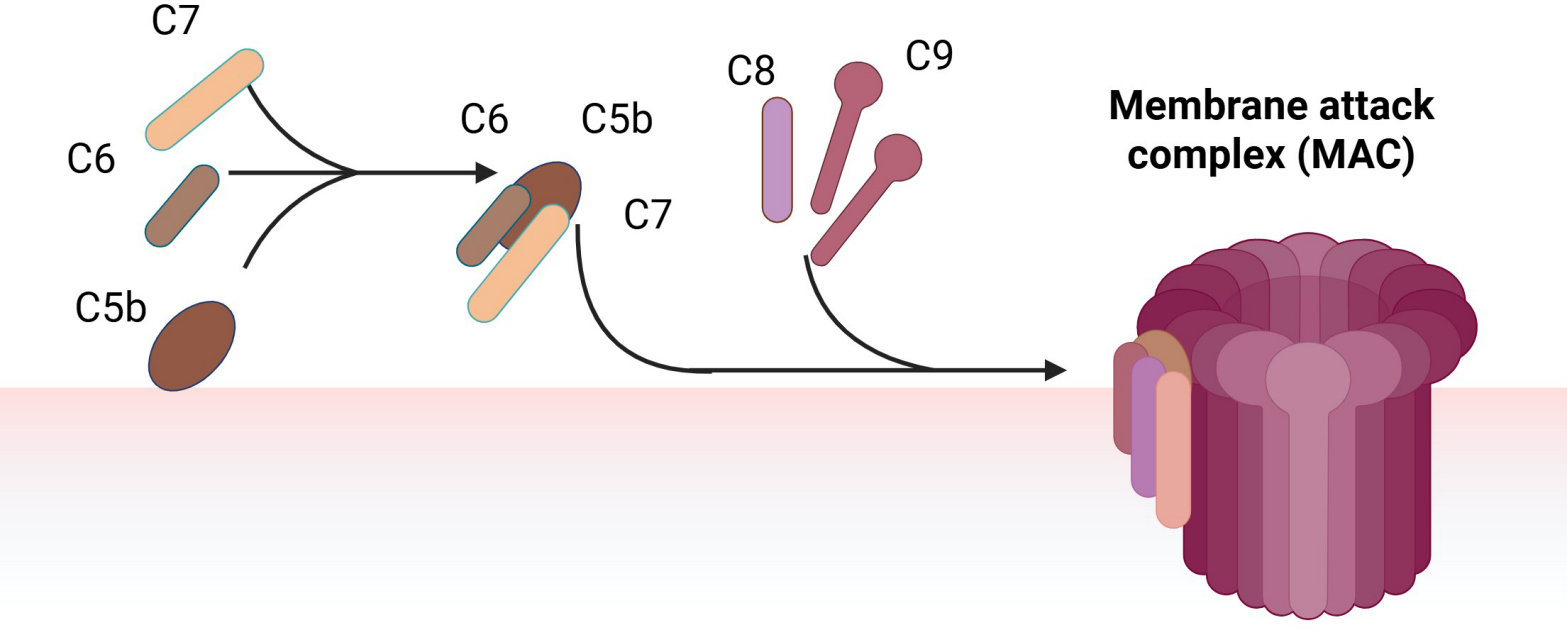
Membrane attack complex formation as part of complement cascade involving C5b and C6-C9.

Due to the limited number of reported cases of cTMA in the context of LT, further investigation of the mechanisms by which complement dysregulation contributes to disease onset may help to improve therapeutic strategies (13). In recent years, targeted inhibition of the terminal complement pathway has emerged as an effective therapeutic strategy for cTMA (14, 15). Eculizumab is a humanized monoclonal antibody that binds to the C5 protein, preventing its cleavage into C5a and C5b, which are essential for the formation of the membrane attack complex (MAC) during inflammation (**Figure 1**) (14, 15). By inhibiting this cleavage, eculizumab effectively disrupts the downstream activation of the complement pathway (15). The use of eculizumab following LT was reported to address vascular rejection episodes in LT recipients. Hereby, the use of eculizumab was regarded safe considering the issue of acute liver injury and levels of transaminases (16, 17). However, the therapeutic management of adult LT recipients who were diagnosed with cTMA shortly after transplantation remains insufficiently characterized. Similar to eculizumab, the long-acting C5-inhibitor ravulizumab was used for the treatment of cTMA (18). Ravulizumab shares a similar mechanism of action but is not yet established as a standardized therapy for cTMA in the post–LT setting.

Here we present a case of cTMA following LT in a patient previously diagnosed with AIH. This case highlights the importance of early recognition of cTMA, careful differentiation from other causes of TA-TMA, and the potential role of complement inhibition as a targeted therapeutic approach in this setting.

## Case presentation

**Figure 2:**
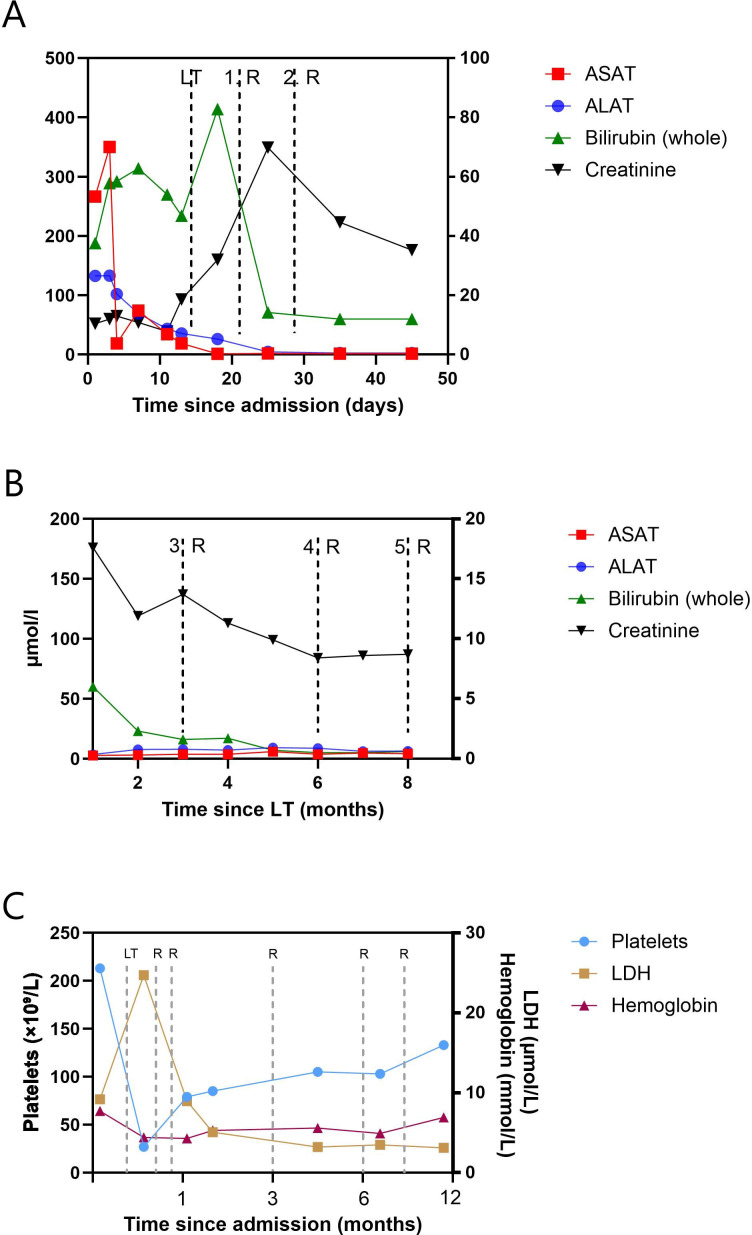
Development of laboratory values after admission including creatinine, bilirubin, AST and ALT (µmol/l). Time of LT, first and second ravulizumab admission are shown **(A)**. Continued development of laboratory values after LT, including creatinine, bilirubin, AST and ALT (µmol/l), three and six months after admission are shown **(B)**. Time course of hemolysis parameters **(C)**, including platelet count, LDH, and hemoglobin levels. Dashed vertical lines indicate ravulizumab administration.

**Figure 3:**
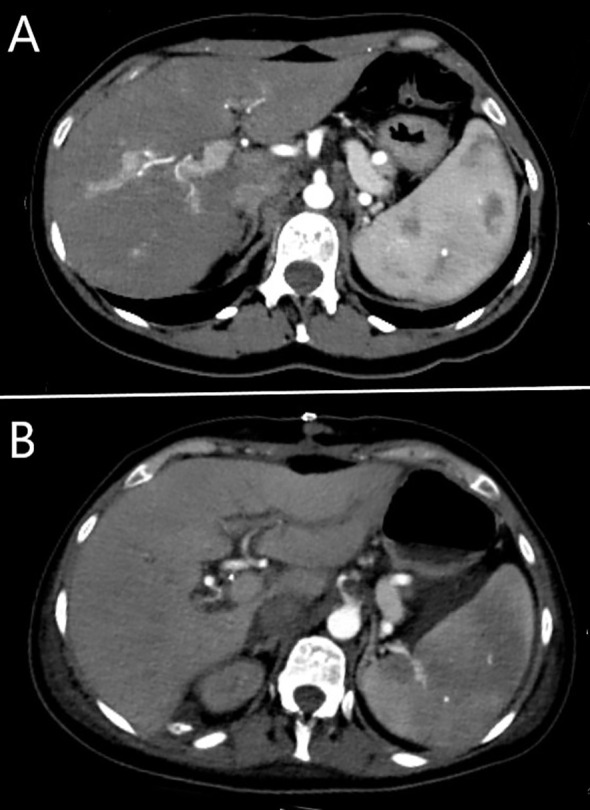
Preoperative computed tomography of the abdomen two weeks after admission to the hospital and occurrence of acute liver injury **(A)**. Postoperative computed tomography of the abdomen after LT **(B)**.

A 36-year-old female patient presented to her family physician with upper abdominal pain and jaundice. She was admitted to our hospital in November 2024. Initial laboratory results were remarkable for elevated liver enzymes, including alanine and aspartate aminotransferase levels (53 µmol/L and 26 µmol/L), and markedly increased direct bilirubin (188 µmol/L). She was admitted to the general ward with a diagnosis of acute liver failure. No underlying cause of liver injury could be identified in her medical history. In addition, no infectious or viral etiology was identified, as hepatitis serology workup, human immunodeficiency virus assay, cytomegalovirus test, and herpes simplex virus assay yielded negative results. After admission, the patient exhibited progressive fatigue accompanied by persistent hyperbilirubinemia, hepatorenal syndrome, and hepatic encephalopathy (**Figure 2A**). Subsequently, the patient was admitted to our intensive care unit and intermittent hemodialysis was started. Computed tomography showed no signs of hepatic cirrhosis or cholestasis (**Figure 3A**).

**Figure 4:**
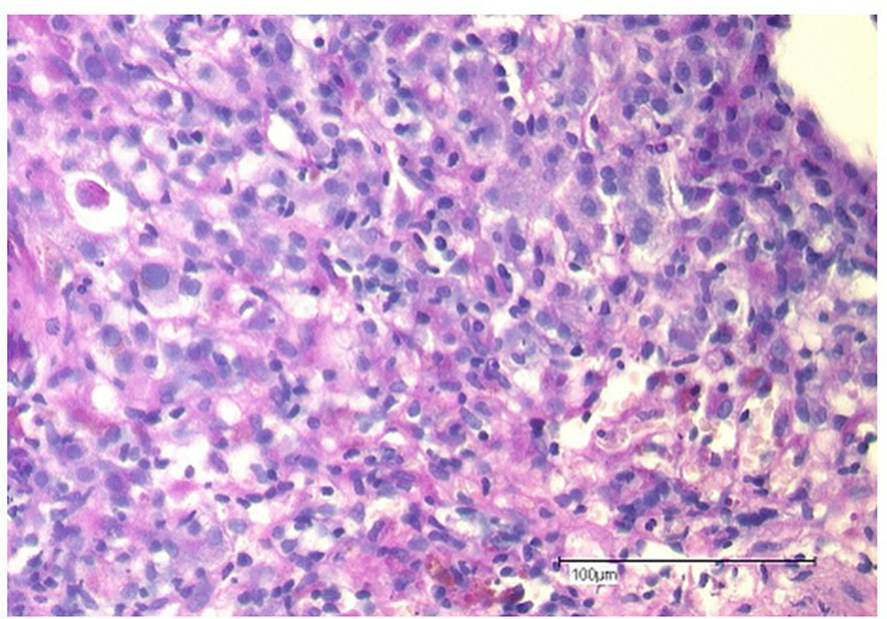
Liver biopsy showing an interface hepatitis, compatible with autoimmune hepatitis in this case.

Despite initiation of glucocorticoid therapy following histological confirmation of AIH, the patient developed progressive liver failure (**Figure 4**). She presented with hepatic encephalopathy and hepatorenal syndrome, requiring admission to the intensive care unit and initiation of intermittent hemodialysis. The diagnosis of fulminant hepatic failure was established according to European Association for the Study of the Liver (EASL) criteria and the patient was evaluated for high urgency transplantation according to the guidelines of the German Medical Association (19). After allocation of a suitable donor organ, LT of a deceased donor was performed. The patient received induction immunosuppression with methylprednisolone, basiliximab, mycophenolate, tacrolimus, and was maintained on tacrolimus, mycophenolate, and prednisone. Tacrolimus levels remained within the therapeutic range during the early postoperative period. Shortly after transplantation, her liver function tests improved compared to baseline. Following LT, liver function improved gradually, however platelet counts continued to decrease (3.6 × 10^4^/mm^3^) and direct bilirubin increased (**Figure 2A**).

On postoperative day 5, laboratory findings demonstrated a rapid decline in platelet count from 213 × 10^9^/L preoperatively to 27 × 10^9^/L, accompanied by a marked increase in lactate dehydrogenase (LDH, 24.7 µmol/L) and a decrease in hemoglobin levels (4.4 mmol/L), consistent with ongoing hemolysis (**Figure 2C**, **Table 1**). Haptoglobin levels were suppressed (<0.1 g/L). Further diagnostic workup confirmed hemolysis with elevated reticulocytes, low haptoglobin, and the presence of schistocytes on peripheral blood smear. Coombs testing was negative, and ADAMTS-13 activity was 64.4%, arguing against severe ADAMTS-13 deficiency. Shiga toxin testing was negative. In addition, heparin-induced thrombocytopenia was excluded by heparin induced platelet activation test and termination of heparin therapy did not result in improvement of platelet count. Concurrently, the patient developed acute kidney injury with anuria requiring continuous renal replacement therapy.

**Table 1:** Hematologic and complement parameters during clinical course.

Parameter	Pre-LT	At TMA diagnosis	After 1st ravulizumab application	After 2nd ravulizumab application	3-month follow-up	6-month follow-up	12-month follow-up
Platelets (×10^9^/L)	213	27	79	85	105	103	133
LDH (µmol/L)	9.2	24.71	8.97	5.06	3.2	3.49	3.11
Hemoglobin (mmol/L)	7.7	4.4	4.3	5.3	5.6	4.9	6.9
Haptoglobin (g/L)	<0.1	<0.1	<0.1	<0.1	<0.1	<0.1	<0.1
C3 (g/L)	n.a.	0.53	1.05	1.51	0.67	1.09	1.3
C4 (g/L)	n.a.	0.16	0.3	0.38	0.19	0.28	0.24
CH50 (U/mL)	n.a.	9.99	9.99	13.2	9.99	9.99	9.99

Values are presented at predefined time points. TMA, thrombotic microangiopathy.

Microbiological analyses, including blood cultures and screening swabs (rectal, nasal, wound, and skin), did not reveal evidence of systemic infection. Apart from rectal colonization with *3MRGN Escherichia coli*, no clinically relevant infectious focus or bacteremia was identified. Inflammatory parameters showed a moderate perioperative increase in CRP, consistent with postoperative systemic inflammatory response.

Based on the constellation of thrombocytopenia, hemolytic anemia, and acute kidney injury, TMA was suspected. The case was discussed in a multidisciplinary setting, including nephrologists, transplant surgeons and hematologists. Given the absence of an identifiable infectious trigger and the lack of response to CNI reduction, classical forms of transplant-associated TMA driven by infection or CNI toxicity were considered less likely. In the context of the clinical presentation and in line with published reports, a complement-mediated mechanism was considered most plausible (**Table 2**).

**Table 2:** Thrombotic microangiopathy after liver transplantation.

Study	Design	LT setting	Entity	Proposed mechanism	Treatment	Key outcome
Nakazawa 2003	Case report	LDLT	TA-TMA	Tacrolimus-associated endothelial injury	Tacrolimus stop + PEX	Improvement
Tamura 2005	Case series (n=10)	LDLT	TA-TMA	Multifactorial endothelial injury with possible complement activation	CNI reduction + PEX	40% mortality rate
Nishi 2006	Case series (n=18)	LDLT	TA-TMA	CNI-related endothelial injury and perioperative inflammatory stress	PEX + CNI change	~56% response
Akamatsu 2007	Case report	LDLT	TA-TMA	Tacrolimus-associated endothelial injury with concomitant CMV infection	CNI switch + PEX	Fatal
Oya 2008	Case report	ABOi LDLT	TA-TMA	Multifactorial endothelial injury associated with ABO incompatibility, CNI exposure, and infection	CNI reduction + PEX + IVIG	Resolution
Matsusaki 2010	Case report	LDLT re-LT	TA-TMA	Tacrolimus-associated endothelial injury	CNI switch + FFP	Resolved
Hori 2011	Case series (n=7)	LDLT	TA-TMA	Severe endothelial and thrombo-inflammatory injury	PEX; 2 re-LT	72% mortality rate
Shindoh 2012	Cohort (n=30 TMA)	LDLT	TA-TMA	Multifactorial endothelial injury with CNI exposure and perioperative sensitization	Multimodal incl. PEX	40-50% mortality rate
Bhatti 2020	Case report	LT	aHUS/cTMA	Complement-mediated endothelial injury suspected	PEX + Eculizumab	Remission
Awidi 2021	Case report	LT	aHUS/cTMA	Complement-mediated TA-TMA suspected	CNI stop + PEX + Eculizumab	Remission

LT, liver transplantation; LDLT, living-donor liver transplantation; ABOi, ABO incompatible; CNI, calcineurin inhibitor; PEX, plasma exchange; IVIG, intravenous immunoglobulin; aHUS, atypical hemolytic uremic syndrome; cTMA, complement-mediated TMA; TA-TMA, transplant-associated TMA.

To assess the suspected cTMA molecular genetic testing was initiated, including Complement Factor H (CFH), CD46, Complement Factor I (CFI), Complement Component 3 (C3), Complement Component 5-9 (C5-9) Factor H-related protein 1 (CFHR 1), Complement Factor H-related protein 5 (CFHR5), Complement Factor B (CFB), Thrombomodulin (THBD), and Diacylglycerol Kinase E (DGKE) (2). The genetic screening showed a heterozygous CFHR1*A haplotype (coding for factor H related protein 1), and a heterozygous C6 VUS. Importantly, no CFHR1 deletion or structural rearrangement was identified.

As differential diagnoses a toxic kidney injury due to hepatorenal syndrome, or postoperative impairment after LT were critically evaluated. Computed tomography was unremarkable for any vascular hepatic abnormalities and showed a regular postoperative status (**Figure 3B**). To evaluate a potential contribution of CNI toxicity, tacrolimus therapy was reduced and subsequently discontinued, with conversion to the mTOR inhibitor everolimus (**Table 3**). This modification of immunosuppressive therapy did not result in improvement of platelet count or hemolysis parameters (**Tables 1**, **3**).

**Table 3:** Inflammatory parameters, immunosuppressive therapy, and microbiological findings.

Parameter	Pre-LT	At TMA diagnosis	After 1st Ravulizumab	After 2nd Ravulizumab	3-month follow-up	6-month follow-up	12-month follow-up
CRP (mg/L)	1.9	30.1	38.3	23.5	1.3	1.1	1.1
Tacrolimus/Everolimus level (µg/L)	7.4*		5.1	5.2	4.9	6.9	5.5
Tacrolimus/Everolimus dosage	2×2.5*	2×2	2×2	2×2	2×2	2×2	2×2
Blood cultures	negative	negative	negative	negative			
Stool culture	not performed	negative	negative	E. coli (3MRGN) STEC/EIEC negative			
Rectal swab	E. coli (3MRGN)	E. coli (3MRGN)	E. coli (3MRGN)	E. coli (3MRGN)			
Nasal swab	no MRSA	no MRSA	no MRSA	no MRSA			
Wound swab	not performed	negative	negative	not performed			
Skin swab	not performed	negative	negative	not performed			

*Initial values under tacrolimus-based immunosuppression before conversion to everolimus.

Subsequently, total plasma exchange (TPE) was initiated (1). After a single session of TPE, the patient received a first dose of ravulizumab followed by another administration of ravulizumab after two weeks. Following initiation of ravulizumab therapy, a rapid and sustained improvement in hematologic parameters was observed, including recovery of platelet count and normalization of LDH levels over time. Hemoglobin levels stabilized in parallel with the resolution of hemolysis (**Figure 2C**). Moreover, bilirubin, AST and ALT decreased (**Figure 2B**). Complement analysis demonstrated decreased C3 and C4 levels at the time of TMA diagnosis, consistent with activation and consumption of the complement system. Additional markers of terminal complement activation, including soluble C5b-9, C5a, C3a, factor H functional assays, and anti-factor H autoantibodies, were not available in the present case.

Following initiation of ravulizumab, both parameters increased and approached the normal range, with subsequent stabilization during follow-up (**Table 1**). CH50 values of 9.99 U/mL represented the lower detection threshold of the assay used at our institution. Persistent suppression of CH50 following ravulizumab administration was interpreted as pharmacodynamic evidence of effective terminal complement blockade at the level of C5. Renal function gradually improved allowing discontinuation of renal replacement therapy ten days after the first administration of ravulizumab. After 33 days of hospital care the patient was discharged with normal liver function and stable renal function. During 12 months of follow-up, ravulizumab was administered every 8 weeks without evidence of TMA relapse. The patient did not experience any treatment-related adverse effects.

## Discussion

By presenting this case, we demonstrate that cTMA can occur as a rare but severe complication following LT, affecting both graft and renal function. Treatment with the C5 antibody ravulizumab after a single session of TPE was associated with recovery of renal function and subsequent improvement in liver function. Thrombotic microangiopathy after LT is typically multifactorial and most commonly associated with CNI toxicity, perioperative endothelial injury, or infection. In contrast, cTMA represents a distinct entity characterized by dysregulation of the alternative complement pathway. Differentiating these entities is clinically relevant, as it directly impacts therapeutic decision-making.

In this view, LT represents a state of profound innate immune activation characterized by ischemia-reperfusion injury, endothelial glycocalyx disruption, cytokine release, and activation of complement-associated inflammatory cascades (3, 4). Endothelial injury may promote activation and amplification of the alternative complement pathway on microvascular surfaces, thereby contributing to endothelial dysfunction and microvascular thrombosis (20). Endothelial injury and complement dysregulation are increasingly recognized as central mechanisms contributing to transplant-associated microangiopathy (7). Recent experimental data further suggest that endothelial glycocalyx disruption may impair complement factor H binding on endothelial surfaces and promote dysregulation of the alternative complement pathway in the setting of calcineurin inhibitor exposure, potentially enhancing complement-mediated endothelial injury (6).

In the present case, infection-associated TMA was considered unlikely, as microbiological investigations did not reveal evidence of systemic infection. Although rectal and intestinal colonization with *3MRGN Escherichia coli* was detected, this finding reflects colonization rather than invasive infection and is not considered a trigger of cTMA. In addition, inflammatory markers, including CRP, were moderately elevated in the perioperative period, which is a well-recognized phenomenon following LT and may reflect systemic inflammatory response rather than infection. This interpretation was supported by consistently negative blood cultures (**Table 3**).

Similarly, isolated CNI-induced TMA was considered but deemed less likely, as tacrolimus levels remained within the therapeutic range. In addition, tacrolimus reduction followed by conversion to everolimus did not result in improvement of thrombocytopenia or hemolysis parameters. A central mechanism underlying cTMA is the dysregulation of the alternative complement pathway, mostly driven by genetic alterations affecting complement regulatory proteins, particularly CFH and related proteins (21). CFH plays a critical role in controlling complement activation on endothelial surfaces by inhibiting the formation and promoting the decay of the C3 convertase. Mutations or structural variants in CFH and CFH-related proteins, including CFHR1 and CFHR5, have been associated with increased susceptibility to cTMA by permitting uncontrolled complement activation and subsequent endothelial injury (22).

In the present case, genetic analysis revealed a heterozygous CFHR1*A haplotype and a heterozygous C6 VUS (23, 24) (16, 17). The detected CFHR1*A haplotype should therefore not be interpreted as equivalent to pathogenic CFHR1 deficiency states associated with anti-factor H autoantibody–mediated aHUS/cTMA. CFHR1 deletions, particularly in the homozygous state, have previously been linked to impaired regulation of the alternative complement pathway and the development of anti-factor H autoantibodies. In contrast, the CFHR1*A haplotype identified in the present case represents a relatively common polymorphic variant reported in the general population and is not considered pathogenic in isolation (25).

Importantly, the identified C6 variant should be interpreted as a VUS. Likewise, C6 is currently not established as a risk gene for aHUS/cTMA, and the pathogenic significance of the detected C6 VUS remains unresolved. No functional validation, segregation analysis, population allele frequency assessment, or ACMG-based pathogenicity classification was available in the present case. Therefore, the identified C6 VUS should not be interpreted as causal but rather as a finding of uncertain clinical relevance.

Anti-factor H autoantibodies were not assessed in the present case. This represents an important limitation, as anti-factor H autoantibodies may provide a mechanistic link between complement dysregulation and endothelial injury in selected patients with cTMA. In addition, plasma exchange may transiently improve disease activity through removal of circulating autoantibodies and complement-associated factors. However, no archived serum or plasma samples suitable for retrospective complement analyses were available.

The genetic findings should instead be interpreted within the broader clinical context, where perioperative endothelial injury, major surgery, systemic inflammation, and immunosuppressive therapy likely contributed to the development of clinically manifest TMA. In this setting, complement-related genetic variants may potentially act as susceptibility factors rather than independent disease-causing mutations. Importantly, rare complement-related variants may also be detected in unaffected individuals from the general population, highlighting the need for cautious clinical and pathophysiological interpretation of genetic findings in cTMA (25).

While C6 is a component of the terminal complement cascade and contributes to membrane attack complex (MAC) formation, there is currently insufficient evidence to establish a direct association between C6 variants and the pathogenesis of cTMA. The multifaceted interplay between complement dysregulation, perioperative endothelial injury, immunosuppressive therapy, and possible genetic susceptibility following LT raises important questions about the role and interpretation of complement genetic testing in cTMA (23, 25, 26). Importantly, genetic findings such as VUS should be interpreted cautiously and within the broader clinical context.

An additional limitation of the present report is the incomplete characterization of complement activation. Although decreased C3 and C4 levels together with the favorable clinical response to C5 inhibition supported the clinical suspicion of cTMA, additional markers of terminal complement activation including soluble C5b-9, C5a, C3a, factor H functional assays, and anti-factor H autoantibodies were not available. Consequently, terminal pathway activation could not be comprehensively characterized on a mechanistic level. Moreover, interpretation of complement parameters is complicated by the administration of TPE prior to initiation of ravulizumab, as plasma exchange may substantially alter circulating complement concentrations and functional assay results.

Taken together, these findings support a multifactorial pathogenesis of cTMA involving perioperative endothelial injury, innate immune activation, pharmacological triggers, and potential complement susceptibility. The underlying diagnosis of AIH may have further contributed to the immunological milieu preceding transplantation. Although AIH is not classically considered a complement-mediated disease, immune dysregulation and alterations in complement activation have been described in autoimmune liver disorders (27). In the present case, pre-transplant complement parameters were not available, limiting the assessment of whether complement dysregulation may already have been present prior to LT. In addition, the transplanted liver may have been exposed to a pro-inflammatory and complement-activating host environment during the early postoperative phase. It is important to note that a diagnosis of cTMA cannot be definitively excluded even when complement levels are within normal ranges or when genetic testing yields negative results (2, 28). A comprehensive genetic screening should focus on a minimum panel of specific genes that were recently reported: CFH, CFHR1, CFHR5, CD46, CFI, C3, CFB, THBD, and DGKE (2).

A review of the literature demonstrates that most reported cases of TMA after LT are classified as TA-TMA, whereas complement-mediated forms remain rare (12, 16). However, increasing evidence suggests that complement dysregulation may play a role in selected cases, particularly in patients with atypical clinical courses or lack of response to conventional therapy (2, 22, 28). Earlier treatment strategies for TA-TMA following LT primarily included modification of immunosuppressive therapy and plasma exchange, with variable outcomes and, in severe cases, the need for retransplantation (12, 15, 17). In contrast, targeted complement inhibition with C5 inhibitors such as eculizumab and ravulizumab represents a more mechanism-based therapeutic approach (15, 18).

In the present case, administration of ravulizumab following a single session of plasma exchange resulted in rapid and sustained improvement of renal function. In addition, the time course of hemolysis parameters, including platelet count, LDH, and hemoglobin levels, demonstrated resolution of hemolysis following initiation of ravulizumab, while complement parameters (C3 and C4) showed initial consumption with subsequent normalization. Persistently suppressed CH50 values after ravulizumab administration were expected and interpreted as pharmacodynamic evidence of effective terminal complement blockade at the level of C5. Ravulizumab has emerged as an effective therapeutic option for cTMA by inhibiting the cleavage C5, thereby preventing the formation of the MAC that contributes to the pathogenesis of the disease (24). It was previously demonstrated that early initiation of eculizumab therapy, a precursor of ravulizumab, at the time of diagnosis is associated with significantly improved clinical outcomes (12, 15, 17). Patients receiving this treatment often experience a reversal of organ damage and an enhanced quality of life. This underscores the importance of initiating treatment for cTMA promptly (14).

In conclusion, this case highlights the importance of considering cTMA in the differential diagnosis of TA-TMA following LT and underscores the potential role of complement inhibition as an effective treatment strategy in selected patients. The multifaceted interplay between complement dysregulation, perioperative endothelial injury, immunosuppressive therapy, and possible genetic susceptibility following LT raises important questions about the role and interpretation of complement genetic testing in cTMA (1, 25). Further studies incorporating comprehensive complement profiling and functional analyses are needed to better characterize complement activation in post-transplant cTMA.

## Data Availability

The original contributions presented in the study are included in the article/supplementary material. Further inquiries can be directed to the corresponding author.
